# Experimental Research on the Wear Behavior of Materials Used in the Manufacture of Components for Cement Concrete Mixers

**DOI:** 10.3390/ma16062326

**Published:** 2023-03-14

**Authors:** Adrian Niță, Marius Gabriel Petrescu, Teodor Dumitru, Andrei Burlacu, Maria Tănase, Eugen Laudacescu, Ibrahim Ramadan

**Affiliations:** Mechanical Engineering Department, Petroleum-Gas University of Ploiești, 100680 Ploiesti, Romania

**Keywords:** wear, concrete mixer, blades, Baroid tribometer, experiment

## Abstract

Mixers used in the production of cement concrete operate under special conditions such as mechanical stresses, abrasive–erosive friction phenomena, and corrosive working environments. In this paper, the authors aimed to establish a correlation between the chemical composition of mixer blade materials and their wear behavior. Three types of alloyed (chromium) cast iron were used for an experimental program that included three sets of tests in accelerated wear conditions which replicated the actual working environment (mixture of mineral aggregate, sand, cement, and water). The tribological tests were carried out using a Baroid tribometer. The results indicated that regardless of the test environment, cast iron with the highest chromium content exhibited the best wear resistance. However, it cannot be concluded that the wear resistance of the studied cast iron materials increases as a direct result of an increase in chromium content. For a chromium content of less than 25%, a better tribological behavior was observed for cast iron with a lower chromium content (of about 4%) than for cast irons with a higher chromium content (of about 9%).

## 1. Introduction

The mixer used in concrete plants is subjected to harsh working conditions when ensuring the mixing of components that go into the production of concrete. The service life of the mixer components (especially the mixing arm blades and the armor plating of the mixing tank walls) depends on the size and nature of the aggregates, the degree of acidity of the cement dust, and the proportion of sand in the aggregate [1,2,3,4,5].

The wear mechanism of a concrete mixer is difficult to control because the wear of the equipment results from the impact and abrasion of a mixture of particles—some of the very small sizes (sand, cement, and adhesives) and others of larger sizes (the aggregates)—in the presence of water, along with the corrosive wear caused by the acidity of the concrete. Therefore, erosion wear specific to the mixture of small particles is corroborated with degradation caused by the impact of large particles on the active elements.

The level of mechanical stress, according to the results of various researchers [6,7,8,9], mainly depends on the hardness of the mixer’s wetted parts, the impact velocity, and the angle of impact.

Various researchers [10,11,12,13,14,15] have studied the operating behavior of mixer components and the mechanical stresses specific to mixer arms and blades.

The scientific literature [6,14,15] has analyzed thestress states in the arms and blades of mixers and has primarily used them as an indicator for evaluating abrasive wear due to impact and friction with aggregates.

Abrasive wear poses significant challenges for the concrete mixer industry, occurring in both dry and wet conditions. The rotating elements are typically made of steel, and the abrasion resistance of the steel determines the lifetime and mass of the drum. In [16], the author designed an experimental stand to test the relative wear resistance of different types of steel under conditions simulating concrete mixers. The tests used crushed granite of 16–25 mm and focused on sliding wear and impact wear for 30 different types of steel [16].

The experiments described in [16] used a mixture of coarse aggregates and water. The results showed that wear occurs primarily on the external surface, so the materials must ensure wear resistance, as the properties of the interior do not significantly affect the equipment’s behavior.

For mixer blades, research has been focused on studying the effect of aggregate particle size and impact angle on the mechanical stresses to which the blades are subjected.

In [17], the wear resistance of blades in planetary concrete mixers was studied, and a new blade shape design that improves the mixer’s performance was proposed. The authors used a combination of experimental testing and numerical simulations to analyze the wear behavior of different blade geometries and to evaluate the performance of the new design.

The results of the study showed that the proposed new blade design significantly reduced wear on the blades compared to the standard blade shape. The authors found that the improved blade shape could reduce the formation of cracks and wear patterns, increasing the overall durability of the mixer. Furthermore, the numerical simulations were in accordance with the experimental results, indicating that the simulation model can accurately predict the wear behavior of mixer blades.

Valigi et al. [18] investigated the wear resistance and efficiency of blades in planetary concrete mixers. The study was performed using a new design for mixing blades to compare with the traditional design. The authors used a 3D simulation model to validate the new blade design and evaluate its mixing efficiency. They also conducted laboratory tests to compare the wear resistance between new and traditional blades. The results showed that the new blade design was better than the traditional one in terms of wear resistance and mixing efficiency.

The scientific work in [19] presents the results of an experimental investigation into a new type of blade for planetary concrete mixers, to assess the wear resistance of this new blade design compared with traditional blades. The newly designed blade showed better wear resistance, and this is significant, since wear is a major issue in concrete mixing, leading to increased maintenance costs and downtime.

The investigation in [20] also focuses on the wear of blades in planetary concrete mixers. The authors used a 3D optical scanner to evaluate wear rates and validate a new blade design. Two blade shapes were tested with the same conditions to compare wear rates and the new design’s effectiveness in reducing wear. The worn blades were measured with a 3D scanner to validate the new design. It was shown that the new design had a 3% lower wear rate and better discharge time than the standard blade, making it more efficient.

The scientific literature contains limited information on the wear characteristics of concrete mixers, where wear occurs not only on the mantle but also on the arms and blades.

The equipment used in the manufacture of concrete on an industrial scale is designed for intense operational loads, mainly represented by the abrasive action of the mineral aggregates (sand and stone of different granulations) and the corrosive action of the mixture of water and cement dust.

The materials used to make the active elements of the equipment are often expensive, and the manufacturing technology is specific to these applications. This is also the case for mixers in asphalt plants, whose component parts—particularly the walls and mixing blades, together with the arms on which they are mounted—are made from special materials that require special maintenance, with decisive replacement intervals for scheduling maintenance activities for the entire plant.

This work aims to provide information on how to choose the materials used in the manufacture of mixing blades, based on their tribological behavior.

## 2. Research Aims, Scope, Motivation, and Novelty

Concrete manufacturers are interested in increasing production efficiency. This objective can be achieved by considering several aspects, such as:

- The use of high-quality raw materials capable of ensuring superior homogeneity of the mix and uniformity of the physical and mechanical characteristics;
- Reducing manufacturing times, which means speeding up technological processes by using substances such as additives or catalysts;
- Optimizing maintenance work on equipment based on the control of wear processes and the appropriate choice of materials used in their manufacture.

While the first two actions involve investment that brings additional costs from the raw materials component, the third action is based on a correct choice of components for the equipment used in the manufacture of concrete, following the evolution of their wear over time. Analyzing the technological flow specific to concrete manufacturing, it can be seen that the mixer is subjected to the most severe loading, and its components operate in highly abrasive environments, which also have a corrosive element. While the materials commonly used in the manufacture of mixing equipment are corrosion-resistant, the abrasion determines the appropriate choice of these materials.

The research conducted in this work focused on the mechanical abrasive/erosive effect produced by cement concrete components on the materials used to plate the mixer’s blades and bowls. In practice, the chromium-alloyed cast irons are used for such equipment, in different variants, the most common being with 4%, 9%, and 25% Cr.

The novelty of the study is given by the experimental program using the most common cast irons used in industry, which included accelerated tests in laboratory conditions in order to investigate the intensity of wear phenomena of mixing blades in an abrasive/erosive environment. Cement manufacturers can, therefore, use the results of this work to make important decisions when choosing the right materials for efficient operating behavior in optimal technical–economic conditions.

It was found that the use of 9% Cr cast iron is not justified because it is more expensive and behaves similarly to the 4% Cr cast iron.

A more expensive version with 25% Cr can offer a reduction in maintenance costs over long periods of use through more frequent blade replacement.

Another advantage of the 25% chromium cast iron is the reduction in downtime costs that can be caused by the need to replace worn parts.

## 3. Materials and Methods

The double-shaft mixer studied, located in the concrete station of the company STRABENBAU LOGISTIC SRL, was a twin shaft type, with a capacity of 2000 L (2 m^3^), produced by CM Italy/SICOMA Italy and, according to the manufacturer’s technical sheet, it has the following constructive–functional characteristics:

- Capacity of 2000 L (2 m^3^ of vibrated concrete);
- The bottom of the mixer has a 15 mm thick Ni-Hard cast iron plate (500 HB, minimum hardness) covered;
- The side panel has 15 mm thick Ni-Hard cast iron sections (500 HB, minimum hardness);
- The mixing arms are made of ductile Ni-Hard cast iron (600 HB minimum hardness);
- The mixing blades are made of alloyed white cast iron (with Cr), having the following hardness values expressed in HV0.2 units (the first value is in carbide and the second in a matrix) for the three types of material:
  - 644 and 318;
  - 1632 and 718;
  - 799 and 462.
- The central axis is made of Ni-Hard cast iron (600 HB minimum hardness);
- The mixer is driven by two 45 kW motors.

The mixing strengths of the blades of horizontal shaft mixers depend on both the properties of the mixture and the parameters of the mixing elements. In horizontal shaft mixers, all the blades have the same surface area and are placed at the same distance from the rotation axis so that the tangential speed is the same for all blades. Most of the blades are also inclined at the same angle of the longitudinal axis of the drum, and, therefore, achieve the same active mixing surface. As a result, the mixing strengths are the same for all the blades in the process [18].

The present experimental research focused on the tribological behavior of the materials used in the manufacture of the mixer blades, based on the analysis of the abrasive wear (abrasive–erosive) of the mixer blades, as measured using a Baroid tester.

The tribological research was performed in order to analyze the behavior of the abrasive action of the mineral aggregates from the concrete composition on different types of cast iron used in the manufacture of mixer blades from concrete plants.

**Figure 1:**
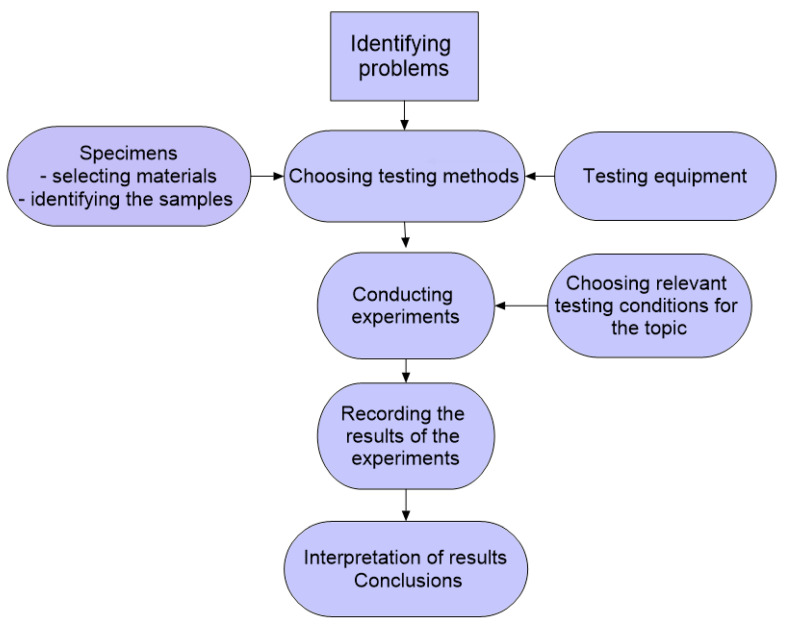
Research program map.

The experimental program went through the following course (Figure 1).

For the tests, three types of alloyed cast iron material (with Cr) were chosen with different Cr contents, namely, 4% Cr, 9% Cr, and 25% Cr.

**Figure 2:**
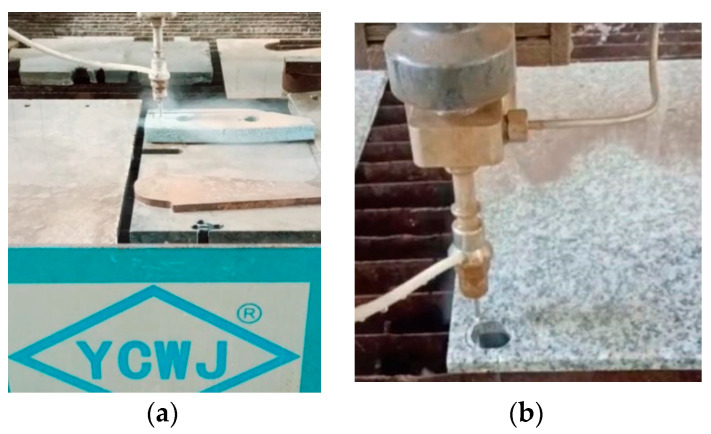

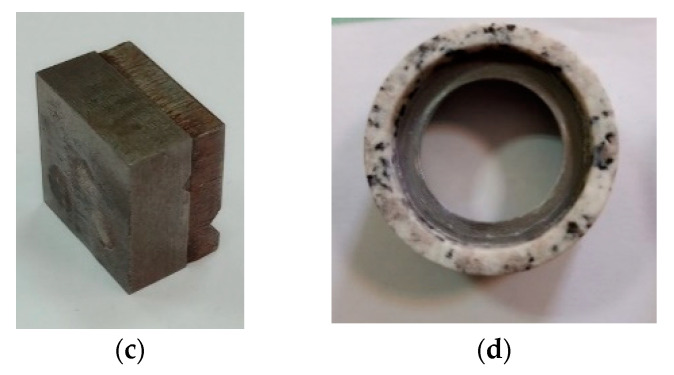
Processing the cast iron samples and the granite rollers: (**a**,**b**)—cutting the samples with the water jet cutting machine; (**c**)—cast iron test sample; (**d**)—granite roller.

The test samples were taken from the blades frequently used at the mixer in the concrete preparation plant. From each blade, corresponding to several types of cast iron, three parallelipipedic samples of 20 mm × 20 mm × 8 mm were taken. To ensure proper fixation of the Baroid-type tribometer, each sample was glued onto a carbon steel support plate, resulting in parallelepipedic samples, as shown in Figure 2.

The test program included three sets of tests for each type of cast iron material. Each set consisted of subjecting the samples to a wear process generated by the friction between their test surface and a rotating granite roller (Figure 2). Granite rollers were used in laboratory tests as they are considered to most faithfully reproduce the friction phenomena that occur between mineral aggregate particles and mixing blades under real conditions. Specific parameters characterized each set of tests, including the test environment, the rotation speed of the granite roller, pressure applied by the roller on the specimen surface, test duration, and the number of test cycles.

Both the cast iron and granite roller specimens were obtained using a water jet cutting process using the WUXI YCWJ-380-X1520 cutting machine (Figure 2) from the Machining Laboratory of the Mechanical Engineering Department within the Faculty of Mechanical and Electrical Engineering. The cutting speed used was 30–40 mm/min for the cast iron samples and 45–60 mm/min for the granite rollers

**Figure 3:**
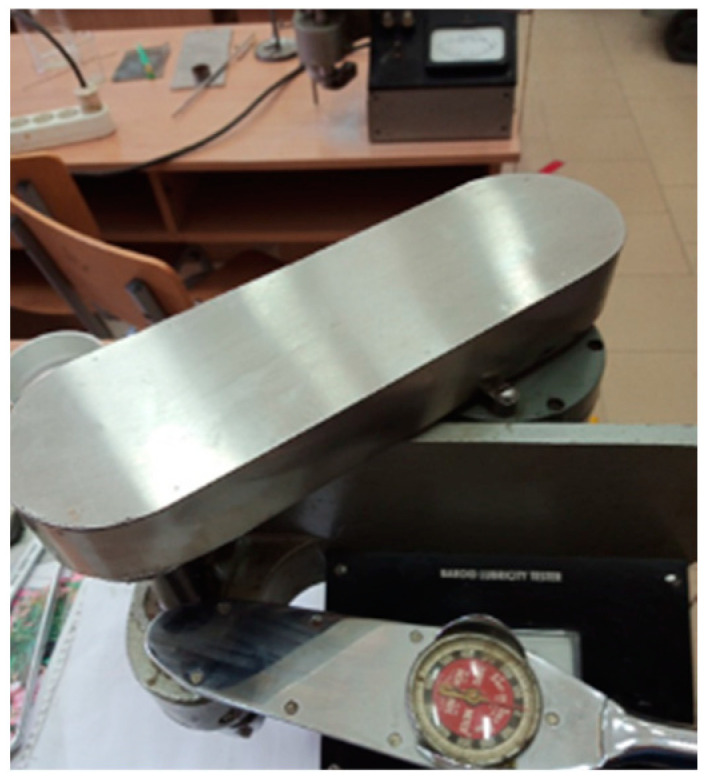
The Baroid tribometer lubricity tester.

A Baroid lubricity tester tribometer (Figure 3) was used to investigate the abrasion and erosion resistance characteristics of the materials, which allowed us to assess their wear behavior, based on mass loss, Δ*m*, of the tested samples.

The operation of the Baroid tester involves the application of a pressing force to the test specimen resulting from the effect of a torque obtained by rotating a lever arm under the action of a predetermined load [21]. Under these conditions, the specimen presses against the test roller (made of mineral material that is similar to the aggregates from the concrete composition) in a rotational motion, thus ensuring the relative movement between the two components of the coupling. The tester offers the conditions for simulating accelerated abrasion/erosion process conditions specific to the movement of mineral aggregates on the surface of the mixer blades, and the results are expressed in gravimetric units. The tester also offers the possibility of measuring the friction oiliness coefficient between the roller and the test sample.

### 3.1. Chemical Composition of the Studied Samples

The blades used in the research were made of three types of cast iron with the chemical compositionsshown in Table 1, as determinedby using the optical emission spectroscopy method performed in the Mechanical Engineering Department. Optical emission spectroscopy, or OES analysis, is a rapid method for determining the elemental composition of a variety of metals and alloys. The equipment used was a Foundry Master Pro from Oxford Instruments.

The results of the microstructure analysis for the three types of material and the hardness measurements can be seen in Table 2. The microstructure of the three samples is specific to white hypoeutectic cast irons, presenting hard constituents in different forms, depending on the Cr content [22,23,24,25].

The weight of the hard constituents in the mass of the examined materials (studying the occupied surface) was determined using the chemical reagent Nital (1–5 cm^3^ acid azotic HNO_3_ and 95–99 cm^3^ ethanol), and the images of the microstructures were processed using ImageJ software after an 8-bit greyscale transformation [26,27,28,29]; the results are presented in Table 2.

### 3.2. Microgeometrical Parameters Determination

**Figure 4:**
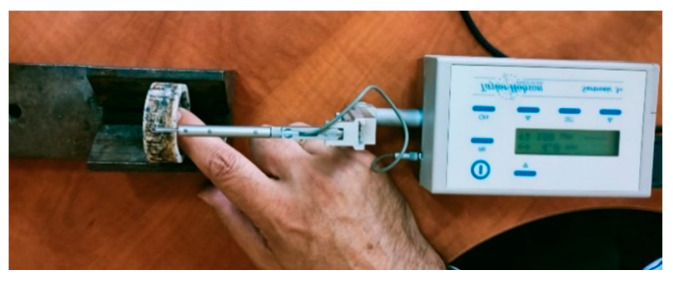
The SURTRONIC 3+ type profilometer—the measurement process.

The research was performed by grinding the surfaces of the specimens on a plane-peripheral grinding machine. To determine the microgeometrical characteristics of the surfaces, the SURTRONIC 3+ profilometer [30] (Figure 4) was used, and the obtained values were processed using the TalyProfileLite 2.1 program. The parameters can be calculated by referencing the asperity profile (*P*), after filtering the roughness profile (*R*), or the waviness profile (*W*). The filter type and reference length are defined for each parameter according to the ISO 4287 standard [31]. Using a Gaussian filter and reference length *l_c_* = 0.8 mm, roughness parameters *R_a_*, *R_t_*, and *R_z_*, bearing curves (Abbott–Firestone), and profilographs for the test specimen surfaces were determined.

**Figure 5:**
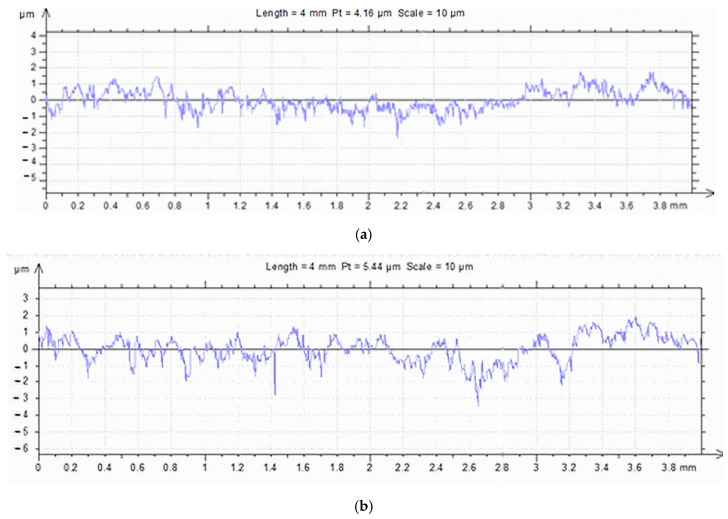

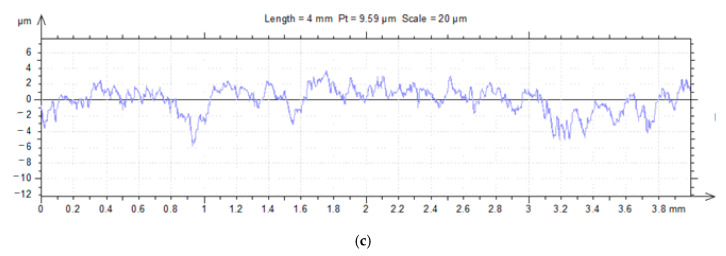
The profile curves for the sample contact surfaces: (**a**) 1/1.2, (**b**) 2/2.1, and (**c**) 3/3.1.

Table 3 shows the profile parameters of the processed surfaces and the measured roughness parameters corresponding to the tested samples. Figure 5 shows the profile curves for samples 1/1.2, 2/2.1, and 3/3.1. 

### 3.3. The Rollers Material Characteristics

The roller’s material quality was in accordance with SR EN 1469:2005, SR EN 12057:2005, and SR EN 12058:2005, as it appears from the manufacturer’s conformity declaration (SC VIGOMARM SRL).

The rollers were made from calc—alkaline granite—whitish gray, with the following mineral composition: feldspars (55–65%)—light gray crystals of albite and plagioclases; quartz (25%); biotite—muscovite (3–5%); amphibole (5–7%); and opaque mineral elements (1–3%, represented by magnetite and ilmenite), and secondary minerals (kaolinite).

The rollers were cut from a semifinished homogeneous product (a plate of 500 × 500 × 10 mm). The surface was smooth, without bumps or unevenness. No deviations from flatness were found.

The roller material had the following physical–mechanical characteristics:

- water absorption at atmospheric pressure—0.38%;
- water absorption by capillarity, mass variation—0.004 g/m^2^·s^0.5^;
- apparent density—2608 kg/m^3^;
- open porosity—0.52%;
- compressive strength—186 MPa;
- flexural strength—15.86 MPa;
- resistance to abrasion (Capone method)—15.85 mm;
- slip resistance: in dry conditions test—99; in wet conditions test—92;
- thermal shock resistance—Δ*V* = 0.01%; Δ*E =* 6.97%.

### 3.4. The Microgeometrical Parameters Determination

**Figure 6:**
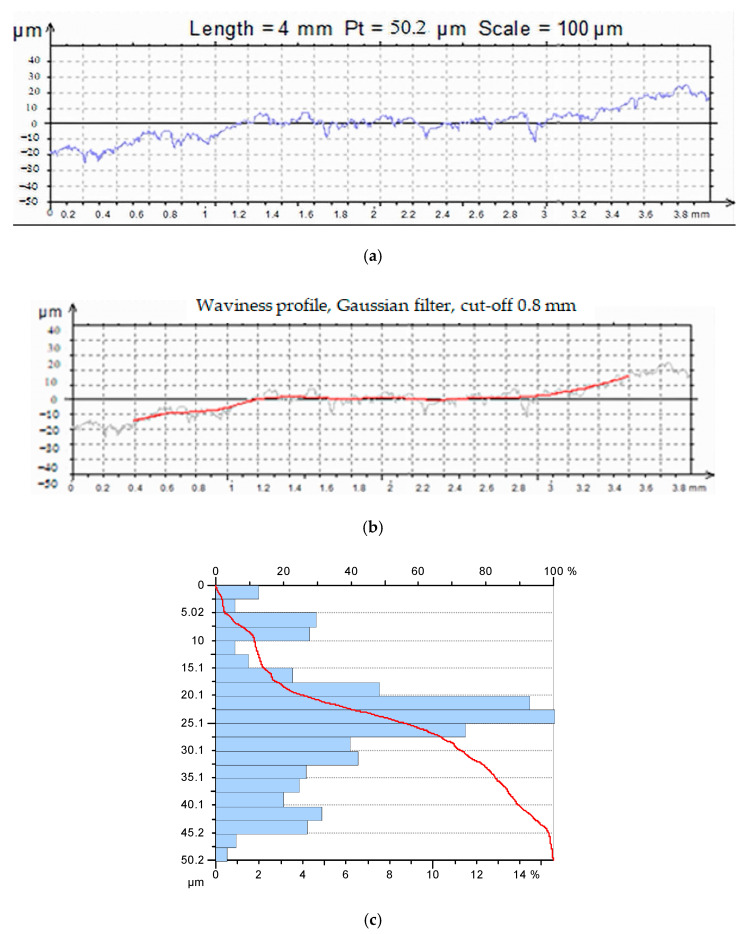
Profile, roughness, and bearing curves for the 3/1 roller: (**a**) Profilogram, (**b**) Profilogram and corrugation, and (**c**) The bearing (Abbott–Firestone) curve.

The granite rollers were similar to the investigated cast iron samples, and the results of the roughness measurements are presented in Table 4. Figure 6 shows the profile, roughness, and lift curves for the 3/1 roller.

## 4. Results and Discussion

The wear of the cast iron samples was determined gravimetrically by weighing at different moments with a KERN ALJ type analytical balance with 10^−4^ g accuracy. For each test, the samples were washed with methylethylketone (MEK), wiped with a solvent-resistant microfiber cloth, and dried in warm air.

To characterize the materials under operating conditions, the experimental program included three sets of tests using different working environments.

I. The first set of tests was characterized by the following conditions:

- working environment—air;
- rotation speed of the granite roller—*n*_roller_ = 60 (±5) rot/min;
- pressing force of the roller on the specimen surface—*F_p_* = 85.5 N;
- testing time—*t_i_* = 30 min;
- number of test cycles—10;
- tested specimens: sample 1/1.2 with roller 3/1; sample 2/2.2 with roller 4/1; and sample 3/3.1 with roller 2/1.

**Figure 7:**
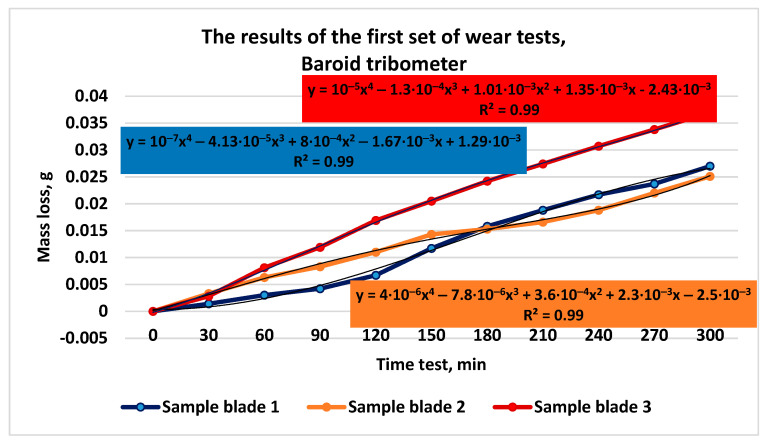
Results of the first set of wear tests.

The comparative results of the mass loss measurements for the three types of tested cast iron are presented in Figure 7.

**Figure 8:**
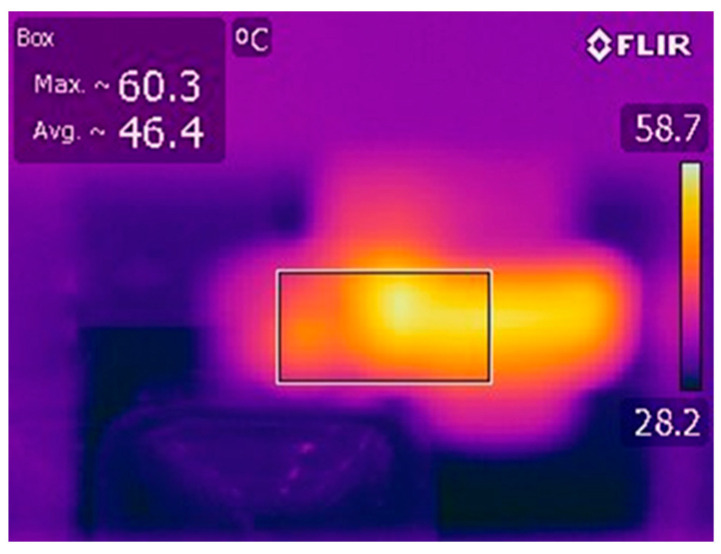
The temperature field in the area of the cast iron sample–granite roller interface during the wear test on the Baroid tester.

The temperatures recorded during the experimental tests (measured using infrared thermography) had relatively high values, as presented in Figure 8.

**Figure 9:**
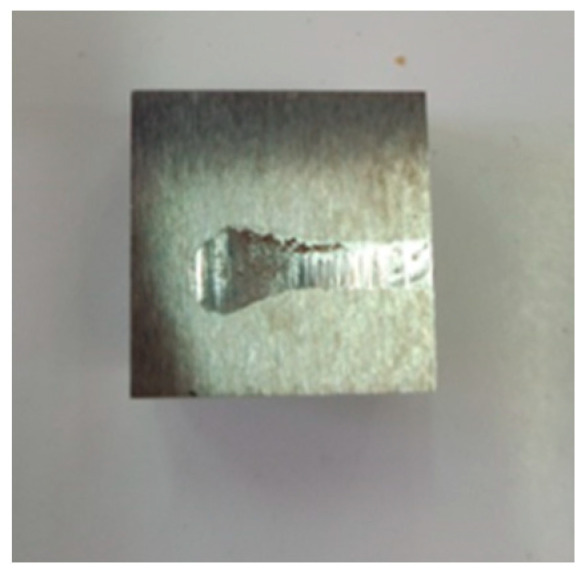
Worn mixer blade sample 2.2/1 (25% Cr) after 240 min—8 cycles of operation.

Figure 9 shows the surfaceof a used specimen for the first set of tests, and the marked traces of abrasive wear can be seen due to the hard stress conditions.

For the first program test (cast iron–roll granite sample, without intermediate environment), the sample was taken from blade no. 3 (made of cast iron alloyed with 9% Cr), which had the lowest wear resistance.

II. The second set of tests was characterized by the following conditions:

- Working environment—a mixture of sand (granulation 0–4 mm) and water in mass proportions of approximately four parts sand and one part water (Figure 9). To maintain similar test conditions, after each test cycle, the working environment was restored (fresh mixture of sand and water);
- Rotation speed of the granite roller—*n*_roller_ = 60 (±5) rot/min;
- Pressing force of the roller on the specimen surface—*F_p_* = 85.5 N;
- Testing time—*t_i_* = 15 min;
- Number of test cycles—10;
- The pH of the mixture at the beginning of a test cycle—7.40 (at an ambient temperature of 25 °C);
- The pH of the mixture at the end of a test cycle—5.09 (at an ambient temperature of 25.5 °C);
- Tested specimens: 1/1.3 sample with 3/2 roller; 2/2.3 sample with 4/2 roller; and 3/3.3 sample with 2/2 roller.

**Figure 10:**
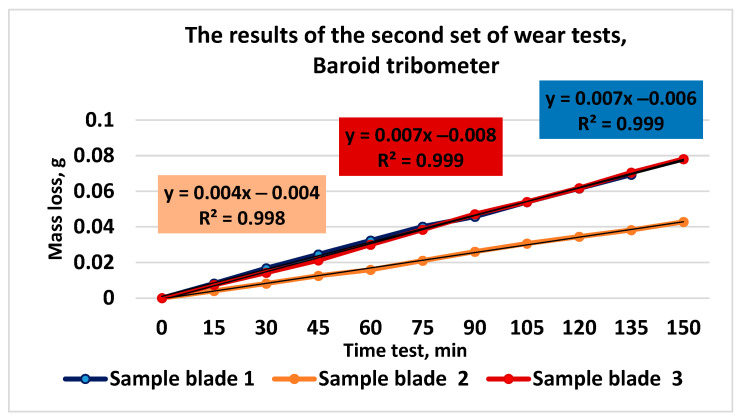
The results of the second set of wear tests.

The comparative results of the mass loss measurements for the three types of tested cast iron are presented in Figure 10.

**Figure 11:**
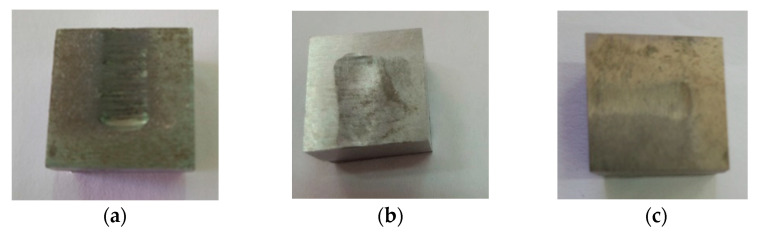
The cross-sectional views of the sample working surfaces at the end of the second set of wear tests: (**a**) worn mixer blade sample 1.2/2 (4% Cr)—working environment of water + sand 0–4 mm (approx. 1:4 mixture ratio) (after 150 min of testing), (**b**) worn mixer blade sample 2.2/2 (25% Cr)—working environment of water + sand 0–4 mm (approx. 1:4 mixture ratio) (after 150 min of testing), (**c**) worn mixer blade sample 2.2/2 (25% Cr)—working environment of water + sand 0–4 mm (approx. 1:4 mixture ratio) (after 150 min of testing).

Figure 11 shows a cross-sectional view of the samples’ surfaces at the end of the second set of wear tests.

**Figure 12:**
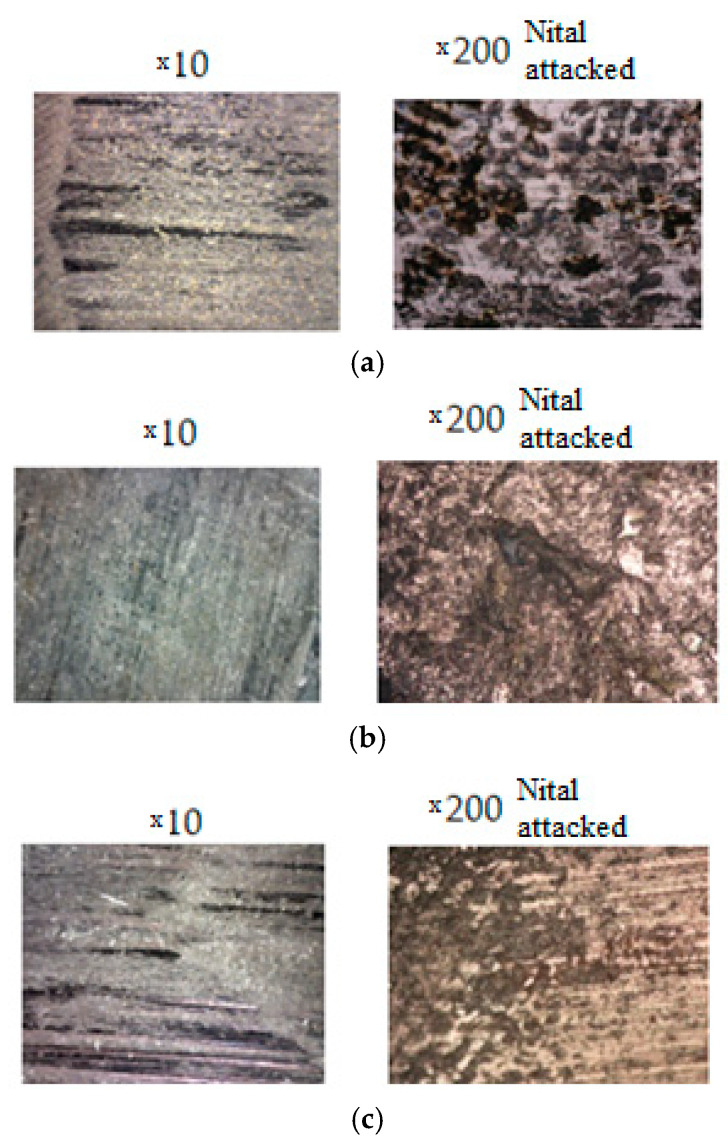
The surface aspect of the samples at the end of the second test program: (**a**) sample 1.2/2; (**b**) sample 2.2/2; and (**c**) sample 3.1/2.

Figure 12 presents the sample surface microstructures in the area with wear, at the end of the second set of tests. Prior to analysis, the samples were polished and chemically etched(attacked) with Nital reagent.

III. The third set of tests was characterized by the following conditions:

- Working environment—cement milk (mixture of cement dust and water in mass proportions of approximately one-part cement dust and 2.75 parts water). The density of the resulting mixture, determined by measurements in the laboratory, was around 1170 kg/m^3^. The cement milk was used to avoid the solidification of the mixture during the testing program. For each sample, a mixture corresponding to the testing environment was made;
- Rotation speed of granite roller—*n*_roller_ = 285 (±10) rot/min;
- Pressing force of the roller on the specimen surface—*F_p_* = 85.5 N;
- Testing time—*t_i_* = 5 min (the test time was reduced to avoid solidification of the mixture during the test program);
- Number of test cycles—9;
- Water pH—7.54 (at an ambient temperature of 30 °C);
- Cement milkpH at the beginning of the test program—11.63 (at an ambient temperature of 30 °C);
- Cement milk pH at the end of the test program—10.84 (ambient temperature of 27 °C);
- Tested specimens: sample 1/1.1 with roller 3/3; sample 2/2.1 with roller 4/3; and sample 3/3.2 with roller 2/3.

**Figure 13:**
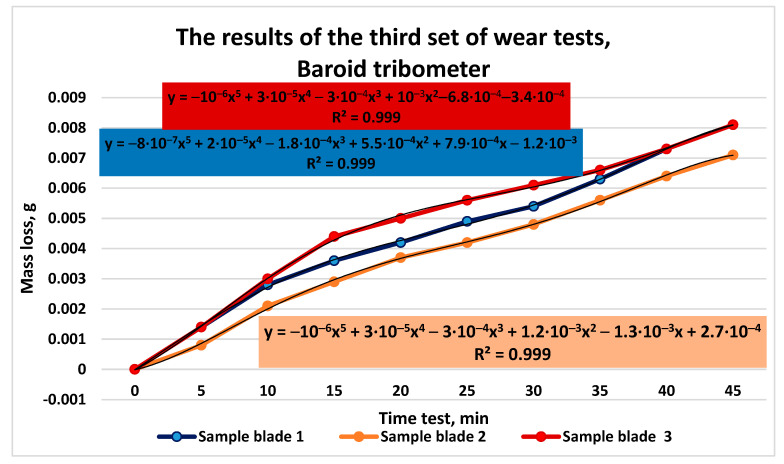
The results of the third set of wear tests.

The comparative results of the mass loss measurements for the three types of tested cast iron are presented in Figure 13.

**Figure 14:**
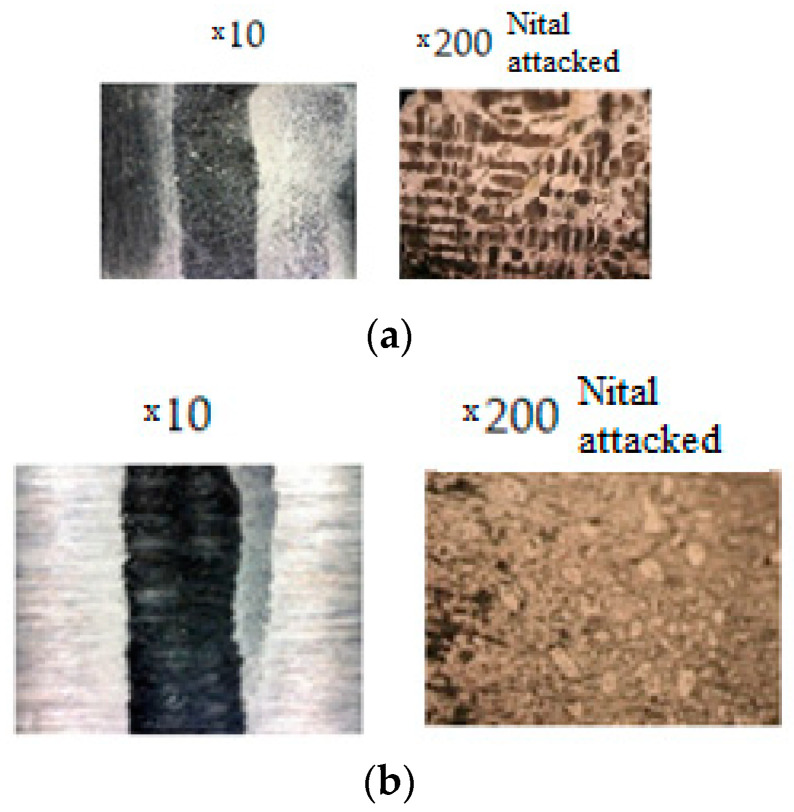

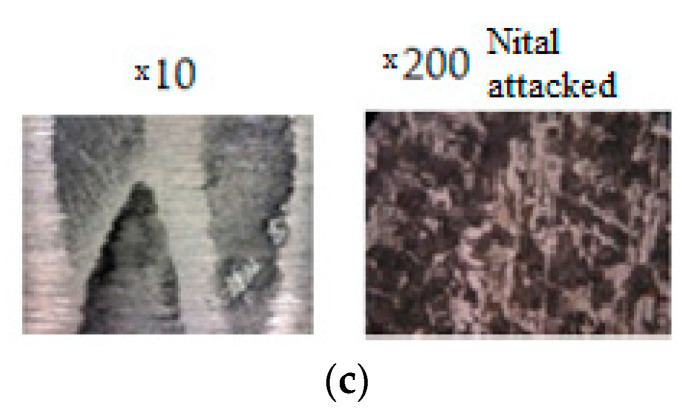
The surface aspect of the samples at the end of the third test program: (**a**) sample 1/1.1; (**b**) sample 2/2.1; and (**c**) sample 3/3.2.

Figure 14 shows the surface aspect of the samples as well as the specimen microstructures in the wear area at the end of the third set of wear tests. Prior to analysis, the samples were polished and chemically etched (attacked) with Nital reagent.

The chemical composition of the three types of cast iron corresponds to the usual chromium-alloyed hypoeutectic cast iron formulations, with chromium contents of 4%, 9%, and 25%. All three types of cast iron studied were classified as white cast iron and they contained carbides with higher hardness than abrasive quartz sand and mineral aggregate grains, as stated in reference [22].

The increased chromium content reduces the risk of cracking and breakage of carbides in the cast iron structure, which undergoes a pronounced abrasion process, according to reference [22]. Therefore, the abrasive material mainly cuts the metal matrix, and the carbide remains on the worn surface for long periods. The wear marks are deep but relatively small in width (see Figure 11).

It can be seen that the samples taken from blade no. 2 (made of highly alloyed cast iron—26% Cr, XCr23 EN-JN3049-type) had the best behavioral performance in the wear tests, in all environments. The increased resistance to abrasion is due to the high chromium content, which is an anti-graphitizing andalphagenic element.

The reduction in the wear rate (mass losses) for sample no. 3 during the experimental programs 2 and 3, and the similar behavior to that of sample no. 1 can be explained by the washing effect of the tested surface (for sample no. 1) and the elimination of abrasive microparticles, possibly embedded superficially.

The similar results obtained for samples no. 1 (4% Cr) and no. 3 (9% Cr) in experimental programs 2 and 3 can be explained by the fact that, for a Cr content between 2% and 10%, the carbides in the structure of the white cast irons are of the (Fe,Cr)_3_C type, ensuring the same wear behavior.

## 5. Conclusions

Analyzing the results of the experimental tests, the following conclusions can be drawn:

- The wear behavior of the three types of cast iron is significantly influenced by the chromium content, an aspect also noted in [22].
- The chromium content has a significant influence on the wear behavior of the three types of cast iron, as previously noted in reference [22].
- The cast iron with 25% Cr provides the best abrasive wear resistance among the tested parts, regardless of the test environment.
- The cast iron with 9% Cr had the lowest abrasive wear resistance in all test environments. When subjected to direct friction with the granite roller without an intermediate environment specific to crushers/stone mills, the 9% Cr samples showed a higher wear rate than the 4% Cr samples.
- In intermediate environments, such as wet sand or milk/cement solution, the wear rates of the two materials are similar, and the 4% Cr sample has better behavior.
- The presence of water, either in combination with sand or in combination with cement, reduces the wear speed for sample no. 3 (made of cast iron alloyed with 9% Cr), obtaining values similar to sample no. 1 (made of cast iron alloyed with 4% Cr).
- The lower wear rate of the 4% Cr sample compared to the 9% Cr sample may be due to the soft matrix that easily incorporates abrasive particles, thus replacing the carbides torn from the metallographic structure, as mentioned in reference [22].
- The carbide in the white cast iron structure with ~2% Cr has the same hardness as abrasive quartz sand, as stated in [22]. Therefore, this value (~2%) represents the critical chromium content required for the transformation of hard abrasives into soft abrasives (by referring to the hardness of the carbides in the cast iron structure).
- The increased resistance to wear of cast irons alloyed with Cr is ensured for Cr percentages above 10%, according to [22]. The usual formulas, in this case, assume a Cr content of 10–35%, which is confirmed by the experimental results obtained for sample no. 2.
- For industrial applications specific to the manufacture of cement concretes, the use of cast iron with 25% Cr or 4% Cr is recommended. The choice of the intermediate grade (9% Cr) is not justified as it has a higher price and lower performance than the 4% Cr grade.

## Figures and Tables

**Table 1:** The chemical composition of the cast iron samples.

Sample	The Chemical Composition, %								
	C	Si	P	S	Cr	Mn	Fe	Ni	Mo
1	3.28	0.76	0.07	0.03	3.83	1.10	89.86	0.89	0.20
2	3.72	0.74	0.02	0.04	25.65	0.87	68.19	0.040	0.35
3	3.08	0.96	0.04	0.03	9.77	1.14	84.12	0.35	0.49

**Table 2:** The microstructures and hardness values for the cast iron samples.

Sample	Sample Microstructure	Hardness HV0.2 (Average of Three Measurements)		Occupied Surface by the Hard Constituents, %	Hardness, Weighted Average
		Matrix	Carbide		
1	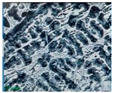 Low cast iron alloyed with Cr (4%), pearlite, ledeburites, and secondary cementites	318	644	44.986	464.654
2	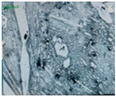 High cast iron alloyed with Cr (25%), austenitic structurewith carbides	718	1632	55.854	1228.505
3	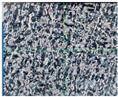 Medium cast iron alloyed with Cr (9%), pearlite, secondary cementite, and ledeburite	462	799	50.739	632.9904

**Table 3:** Roughness values of the cast iron specimens.

Sample/Sample Symbolization	1/1.1	1/1.2	1/1.3	2/2.1	2/2.2	2/2.3	3/3.1	3/3.2	3/3.3
Roughness value *R_a_*, μm	0.555	0.329	0.569	0.452	0.675	0.528	0.875	0.805	0.81

**Table 4:** The granite roller roughness values.

Roller Symbolization	3/1	4/1	2/1	3/2	4/2	2/2	3/3	4/3	2/3
Roughness value *R_a_*, μm	2.17	2.56	2.09	1.79	3.2	2.61	2.10	2.19	2.21

## Data Availability

Data is contained within the article.
